# Comprehensive analysis regarding the prognostic significance of downregulated ferroptosis-related gene AKR1C2 in gastric cancer and its underlying roles in immune response

**DOI:** 10.1371/journal.pone.0280989

**Published:** 2023-01-26

**Authors:** Wei Liu, Fan Zhang, Keda Yang, Yuanliang Yan

**Affiliations:** 1 Department of Pathology, Xiangya Hospital, Central South University, Changsha, China; 2 Department of Orthopedic Surgery, The Second Hospital University of South China, Hengyang, China; 3 Department of Gynecology, Xiangya Hospital, Central South University, Changsha, Hunan, China; 4 Department of Pharmacy, Xiangya Hospital, Central South University, Changsha, China; The First Affiliated Hospital of Nanjing Medical University, CHINA

## Abstract

Ferroptosis is a cell death form that has been reported to be involved in the progression of gastric cancer (GC). However, the underlying mechanism of ferroptosis in GC still needs to be further explored. This study conducted a survey regarding the biological functions of ferroptosis-related gene AKR1C2 in GC. Multiple bioinformatic platforms were applied to indicate that the expression level of AKR1C2 was downregulated in GC tissues, which displayed good prognostic value. Clinical statistics proved that AKR1C2 expression was correlated with several tumor characteristics of GC patients, such as characteristics of N-stage tumor or residual tumor. Additionally, LinkedOmics was employed to explore the co-expression network and molecular pathways of AKR1C2 in GC. Eventually, AKR1C2 was found to be involved in several immune-related signatures, such as immunostimulators, immunoinhibitors, chemokines and chemokine receptors. To sum up, these results may provide a novel insight into the significance and biological functions of ferroptosis-related gene AKR1C2 in GC tumorigenesis.

## Introduction

Gastric cancer (GC), as one of the most common malignant tumors in the world, ranks the fifth most diagnosed cancer worldwide [1]. Nowadays, several therapeutic strategies have been reported to be effective in the treatment of GC patients, including surgical treatment, chemotherapy, and immunotherapy [2]. However, the survival rate of GC is still low. Therefore, it is high time that we should explore a new biomarker for predicting the prognosis of GC.

Ferroptosis is a regulated cell death form that is strongly related to the accumulation of ferrous iron and the peroxidation of lipid, and it ends up with mitochondrial dysfunction and toxic peroxidation of lipid [3]. Ferroptosis-related treatment could be a promising therapeutic strategy for cancer cells [4]. Emerging studies reported that ferroptosis plays a significant role in eliminating cancer cells. A study identified that P53RRA-G3BP1 could be a new mechanism of promoting the ferroptosis of lung adenocarcinoma [5]. Furthermore, the combination of ferroptosis induction with anti-PDL1 therapy may have synergistic effects on antitumor activities [6]. Additionally, the effects of radiation treatment may be partially correlated with ferroptosis. However, the detailed mechanism of ferroptosis in GC should be further explored and verified.

The aldo-keto reductases family 1 member C2 (AKR1C2) has been reported to cause the mutual translation between sex hormones and associated inactive metabolites. As a member of AKR1C subfamily, the substrate specificity of AKR1C2 is quite different from other AKR1C members [7]. Recently, the significance of AKR1C2 in malignant tumors has been explored. A study demonstrated that the higher expression level of AKR1C2 is correlated with better prognosis in patients with thyroid carcinoma [8]. AKR1C2 combined with SOCS1 could exert synergistic effects on predicting the prognosis in patients with acute myeloid leukemia (AML) [9]. Another study identified that the upregulation of AKR1C2 is strongly correlated with the pathological stage and has worse prognosis in patients with esophageal squamous cell carcinoma (ESCC) [10]. However, few studies have reported the correlation between AKR1C2 and GC patients.

This study will further explore and elucidate the underlying mechanism of AKR1C2 in GC. By using some bioinformatics tools, we found that downregulated AKR1C2 could be a prognostic prediction tool of GC. Additionally, this study investigated the correlation between clinical characteristic parameters and AKR1C2 expression. Then, gene-set enrichment analysis (GSEA) was performed to reveal the biological functions and signaling pathway. To further explore the vital roles of AKR1C2 in immune regulation, we evaluated the correlation between AKR1C2 and tumor-infiltrating immune cells (TIICs) of GC. The above investigations uncovered that AKR1C2 may be taken as a potential predictive prognostic biomarker and it plays a vital role in immunotherapy of GC patients.

## Materials and methods

### Data collection

We searched three GC datasets from Gene Expression Omnibus (GEO) database [11], including GSE26942 [12], GSE112369 [13] and GSE33651 [14] (**Table 1**). Additionally, the differential expressed genes (DEGs) between the normal gastric tissues and GC tissues were analyzed. The cut-off value was set up as follows: p-value < 0.05 and | logFC| ≥ 0.8. Later, Venn analysis was applied to investigate the co-differentially expressed genes (co-DEGs) between ferroptosis-related gene dataset and the above three GEO datasets. As a public database, TCGA database has provided the clinical information concerning 33 types of cancer [15]. Both the gene expression profiles and clinical information of GC patients were retrieved from TCGA database, including 32 adjacent-tumor samples and 375 GC samples.

**Table 1:** The characteristics of three GEO datasets about gene expression profiling by array.

GEO^a^ datasets	Platform	Sample size		DEGs^b^	References
		cancer	normal		
GSE26942	GPL6947	202	12	105 up-regulated genes and 588 down-regulated genes	[12]
GSE112369	GPL15207	36	8	710 up-regulated genes and 1140 down-regulated genes	[13]
GSE33651	GPL2895	40	12	959 up-regulated genes and 143 down-regulated genes	[14]

^a^ GEO, Gene Expression Omnibus datasets.

^b^ DEGs, differentially expressed genes.

### Bioinformatics analysis

Kaplan-Meier plotter was adopted to analyze the prediction value of prognosis of co-DEGs in GC patients. The overall survival (OS), first-progression (FP), post progression survival (PPS) of co-DEGs were downloaded from Kaplan-Meier plotter [16]. Then, we utilized TNMplot [17], GEPIA2.0 [18] combined with the Cancer Genome Atlas (TCGA) to further explore the AKR1C2 gene expression in normal and tumor groups. The TNMplot database could be used to analyze differential gene expression in tumor, normal and metastatic tissues, and it includes RNA-sequence data from TCGA database and gene chip data from GEO database. Then, the correlation between the expression level of AKR1C2 and clinical characteristic parameters, such as T stage, pathologic stage, gender and age, were also identified. Furthermore, we applied LinkedOmics algorithm [19] to analyze the correlation between co-expressed genes and AKR1C2. We downloaded the co-expressed molecules of AKR1C2 in GC samples from cBioPortal database [20] **(S1 Table)**. After that, we used Xiantao tool (https://www.xiantao.love/products) to analyze the Gene ontology (GO) enrichment of AKR1C2 co-expressed genes. Furthermore, we have utilized the STRING [21] to analyze the protein-protein network of AKR1C2. Next, the single-sample GSEA (ssGSEA) [22] was conducted to explore the correlation between AKR1C2 level and 24 types of TIICs in GC. Moreover, the TISIDB [23] and TIMER [24] were employed to further verify the correlation between immune infiltration cells and AKR1C2. Finally, we explored the correlation between AKR1C2 and the immune checkpoints, such as CTLA4 and VSIR.

### Immunohistochemical (IHC) analysis

The GC tissues and normal stomach tissues were collected from the Department of Pathology, Xiangya Hospital, Central South University. IHC technique was used to explore the expression levels of AKR1C2 and VSIR in the tumor group and normal group. The personal data of subjects (such as date of birth, gender, race, ethnicity, health information, etc.) were replaced by coding to protect their privacy and rights. The appropriateness of the design of the study was approved by the Ethical Committee of Xiangya Hospital, Central South University, with the approval number of 202201019.

### Statistical analysis

We utilized SPSS 19.0 software to conduct the statistical analysis. P-value<0.05 was considered to be statistically significant. The survival analysis was developed by using Kaplan-Meier analysis. The expression levels of GC group and normal group were evaluated with t-test. The correlation between AKR1C2 expression and clinical characteristics was analyzed by using R package and logistic regression.

## Results

### Differentially expressed genes between normal and GC groups

We searched the GEO database and downloaded three datasets from it. The p value was set as follows: p-value<0.05 and | logFC| ≥ 0.8, which was used to identify the differentially expressed genes (DEGs) between the GC group and normal tissue group. There are 105 up-regulated genes and 588 down-regulated genes in GSE26942, 710 up-regulated genes and 1,140 down-regulated genes in GSE112369, and 959 up-regulated genes and 143 down-regulated genes in GSE33651 (**S2 Table**).

**Fig 1:**
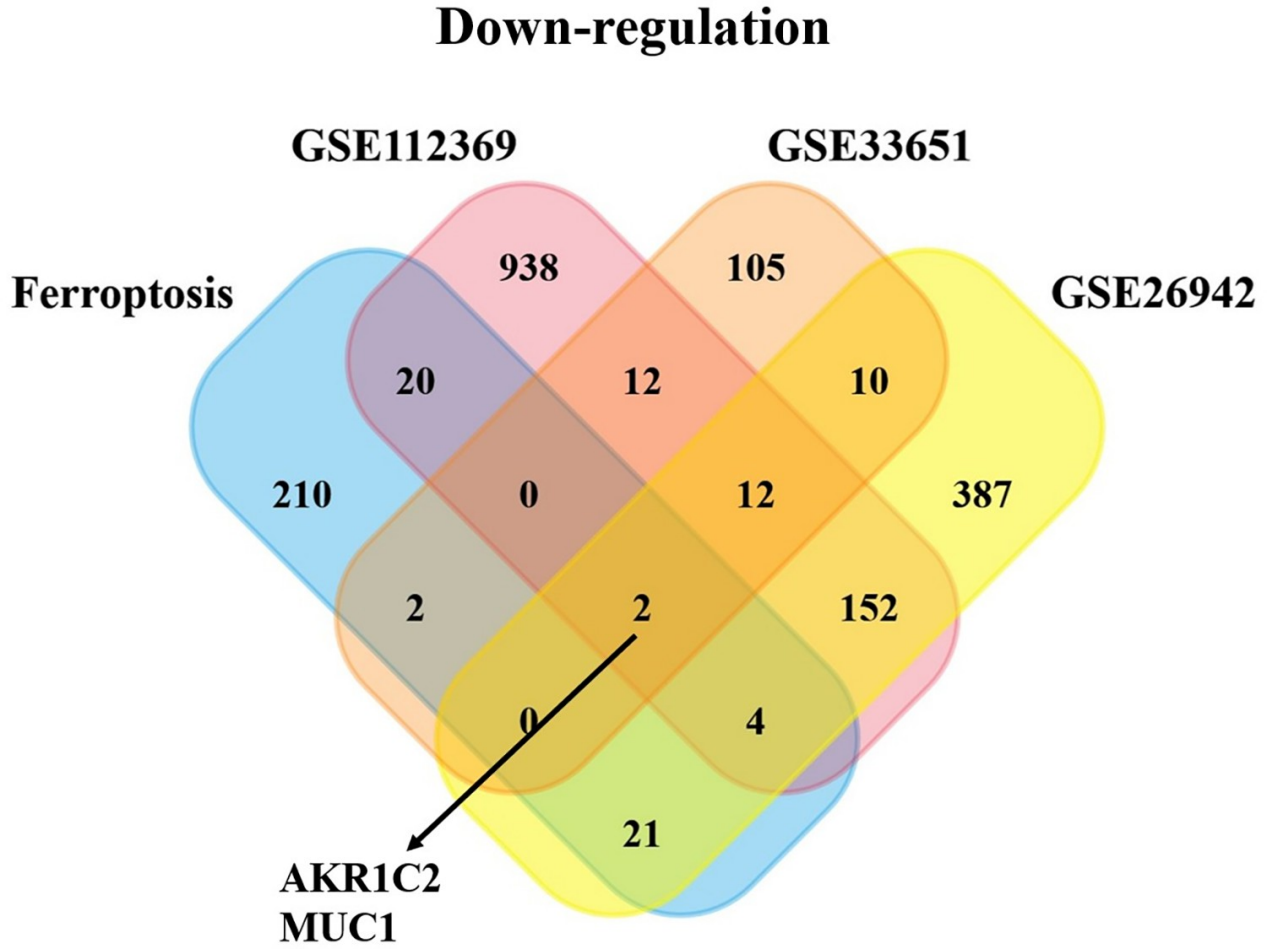
Venn analysis depicted the downregulated co-DEGs between the ferroptosis related gene dataset and the three GEO datasets. There are two downregulated genes in the four datasets, named as AKR1C2 and MUC1.

Moreover, a recent study found that ferroptosis may participate in the development and progression of certain types of tumors [25]. Therefore, it is necessary for us to further explore whether ferroptosis plays a significant role in the process of GC patients. Then, Venn analysis was established to identify co-DEGs between the ferroptosis-related gene dataset and the three GEO datasets. The plot shows that two down-regulated genes AKR1C2 and MUC1 have potential significance in GC patients (**Fig 1**). However, no up-regulated co-DEGs exist between the three GEO datasets and ferroptosis-related gene dataset (**S1 Fig**).

### AKR1C2 offering good prognosis in patients with gastric cancer

**Fig 2:**
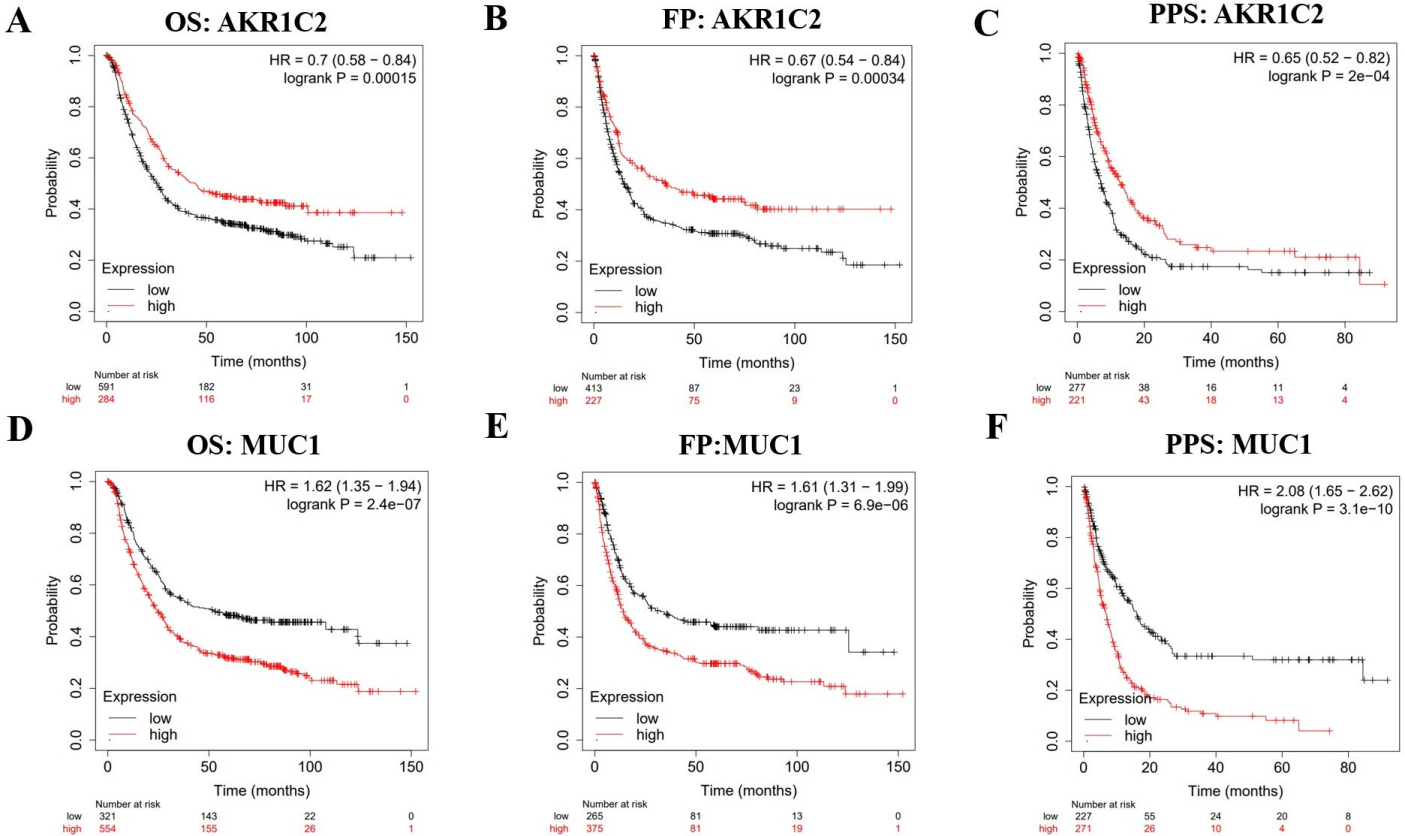
Kaplan-Meier Plotter depicting the prognostic values of AKR1C2 and MUC1 in gastric cancer. **(A-C)** The prognostic values of AKR1C2 in GC patients. **(D-F)** The prognostic values of MUC1 in GC patients. OS: overall survival, FP: first-progression, PPS: post progression survival.

Kaplan-Meier plotter was employed to identify the correlation between the expression levels of AKR1C2 and MUC1 and the prognosis of GC patients, including OS, FP and PPS. The results demonstrated that higher expression level of AKR1C2 was correlated to better OS (HR = 0.7, 95% CI = 0.58–0.84, p = 0.00015), FP (HR = 0.67, 95% CI = 0.54–0.84, p = 0.00034) and PPS (HR = 0.65, 95% CI = 0.52–0.82, p = 2e-04) in all GC patients (**Fig 2A–2C**). Inversely, lower expression level of MUC1 was correlated to better OS (HR = 1.62, 95% CI = 1.35–1.94, p = 2.4e-07), FP (HR = 1.61, 95% CI = 1.31–1.99, p = 6.9e-06) and PPS (HR = 2.08, 95% CI = 1.65–2.62, p = 3.1e-10) in all GC patients (**Fig 2D–2F**). These results indicated that AKR1C2 expression may predicate a good prognosis in GC patients.

### AKR1C2’s lower expression in GC group than normal group and the correlation between AKR1C2 and clinical characteristics

**Fig 3:**
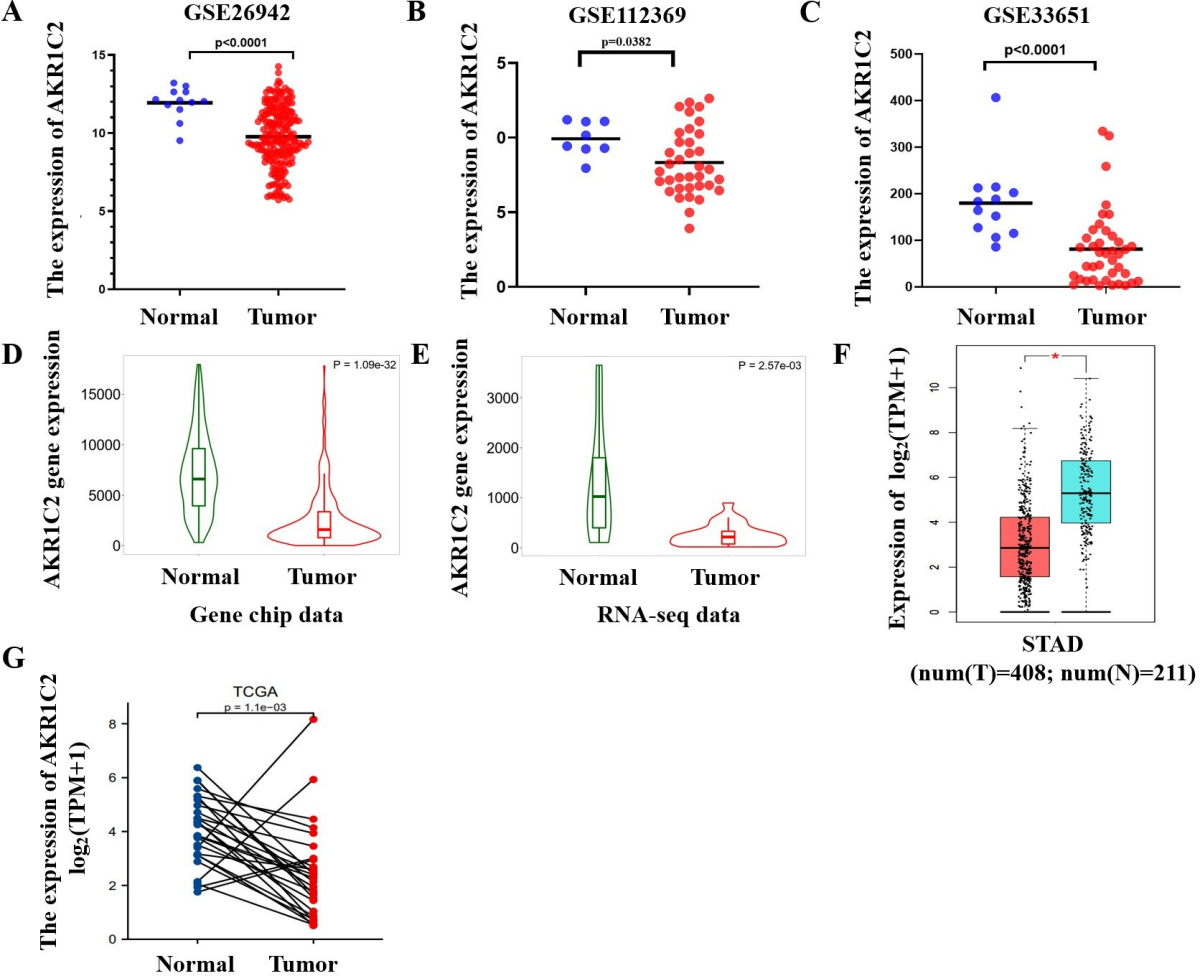
The expression levels of AKR1C2 in GC patients. **(A-C)** In the three GEO datasets, the expression level of AKR1C2 was lower in GC tissues than normal gastric tissues. **(D-E)** The TNMplot database illustrating the expression level of AKR1C2 was lower in GC tissues than normal gastric tissues from gene chip data and RNA-seq data. **(F-G)** The GEPIA 2.0 database and TCGA database depicting AKR1C2 expressed differently in GC group and non-cancerous group. *P < 0.05.

The three GC datasets (GSE26942, GSE112369 and GSE33651) were downloaded from the GEO database. The diagraphs demonstrated that AKR1C2 was expressed much more highly in normal tissues than in GC groups (p<0.05) (**Fig 3A–3C**). Furthermore, the pictures downloaded from the TNMplot database showed that AKR1C2 expression was higher in normal tissues compared to GC tissues from gene chip data (p = 1.09e-32) and RNA-seq data (p = 2.57e-03) (**Fig 3D and 3E**). The GEPIA2.0 database identified that AKR1C2 was expressed highly in normal group (**Fig 3F**). Additionally, the statistics obtained from TCGA database further verified that the expression levels of AKR1C2 in GC tissues and the normal tissues exhibited a difference (p = 1.1e-03) (**Fig 3G**).

Next, we investigated the correlation between AKR1C2 expression and clinical characteristic parameters in GC patients via TCGA database. We found that the mRNA expression level of AKR1C2 was correlated with N stage tumor (p = 0.014) and residual tumor (p = 0.025) (**Table 2**).

**Table 2:** The correlation between the expression of AKR1C2 and clinical characteristic parameters in GC patients from TCGA.

Characteristics	Total(N)	Odds Ratio (OR)	P value
T stage (T3&T4 vs. T1&T2)	367	0.855 (0.538–1.356)	0.506
N stage (N2&N3 vs. N0&N1)	357	1.702 (1.115–2.610)	0.014
M stage (M1 vs. M0)	355	1.756 (0.769–4.256)	0.191
Pathologic stage (Stage III&Stage IV vs. Stage I&Stage II)	352	1.312 (0.863–1.999)	0.205
Gender (Male vs. Female)	375	0.877 (0.574–1.338)	0.543
Age (>65 vs. < = 65)	371	0.907 (0.602–1.367)	0.642
Residual tumor (R2 vs. R0&R1)	329	4.306 (1.356–19.045)	0.025
Reflux history (Yes vs. No)	214	1.264 (0.630–2.556)	0.510
Barretts esophagus (Yes vs. No)	208	2.063 (0.706–6.830)	0.201
H pylori infection (Yes vs. No)	163	0.747 (0.271–1.999)	0.561
Histologic grade (G3 vs. G1&G2)	366	1.023 (0.673–1.555)	0.915

### AKR1C2 co-expression network in gastric cancer

**Fig 4:**
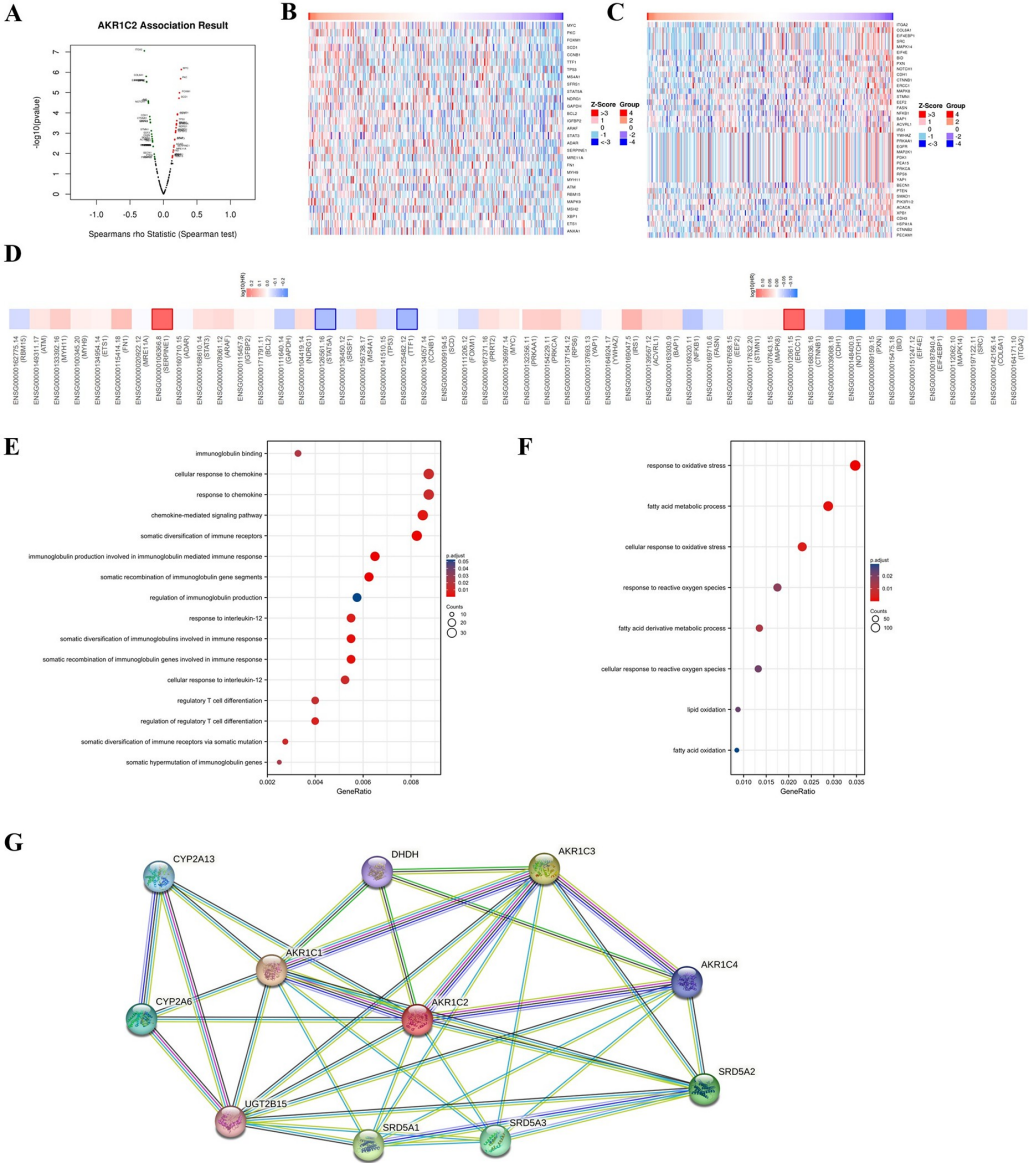
The co-expressed genes of AKR1C2 in GC. **(A)** The LinkedOmics portraying the AKR1C2-related genes in GC. **(B)** The heatmap indicated the top 29 genes that possessing positive correlation with AKR1C2 in GC. **(C)** The heatmap indicated the top 39 genes that possessing negative correlation with AKR1C2 in GC. **(D)** Survival heatmaps illustrating the top 50 genes possessing positive and negative relationship with AKR1C2 in GC. **(E-F)** GO enrichment analysis of AKR1C2 co-expressed molecules in GC patients. **(G)** The protein-protein network of AKR1C2 via STRING database.

LinkedOmics was employed to explore the co-expression gene and biological functions of AKR1C2 in GC patients. As shown in the picture, red dots represent the genes positively associated with AKR1C2 and green dots represent the genes negatively associated with AKR1C2 (p<0.05) (**Fig 4A**). In addition, the heatmap showed that 29 genes were positively correlated with AKR1C2 (**Fig 4B**). **S3 Table** illustrated the top 25 genes that were positively correlated with AKR1C2. There are 39 genes that were negatively correlated with AKR1C2 (**Fig 4C**). Furthermore, **S4 Table** listed the top 20 genes that were negatively correlated with AKR1C2. In addition, among the top 25 positive-correlated genes, AKR1C2 was reported to have significant results in SERPINE1, STAT5A and TTF-1. Among the 20 negatively-correlated genes, AKR1C2 was found to have significant results in ERCC1(**Fig 4D**). The GO enrichment analysis illustrated that the co-expressed genes of AKR1C2 mainly participated in several immune-related pathways, such as chemokine-mediated signaling pathway and somatic diversification of immune receptors, and ferroptosis-related pathways, such as response to oxidative stress and fatty acid metabolic process (**Fig 4E and 4F**). In addition, the STRING database indicated the important association between AKR1C2 and several malignant disease-associated molecules, such as DHDH, SRD5A1 and SRD5A2 (Fig 4G).

### Correlation between AKR1C2 and immune regulation

**Fig 5:**
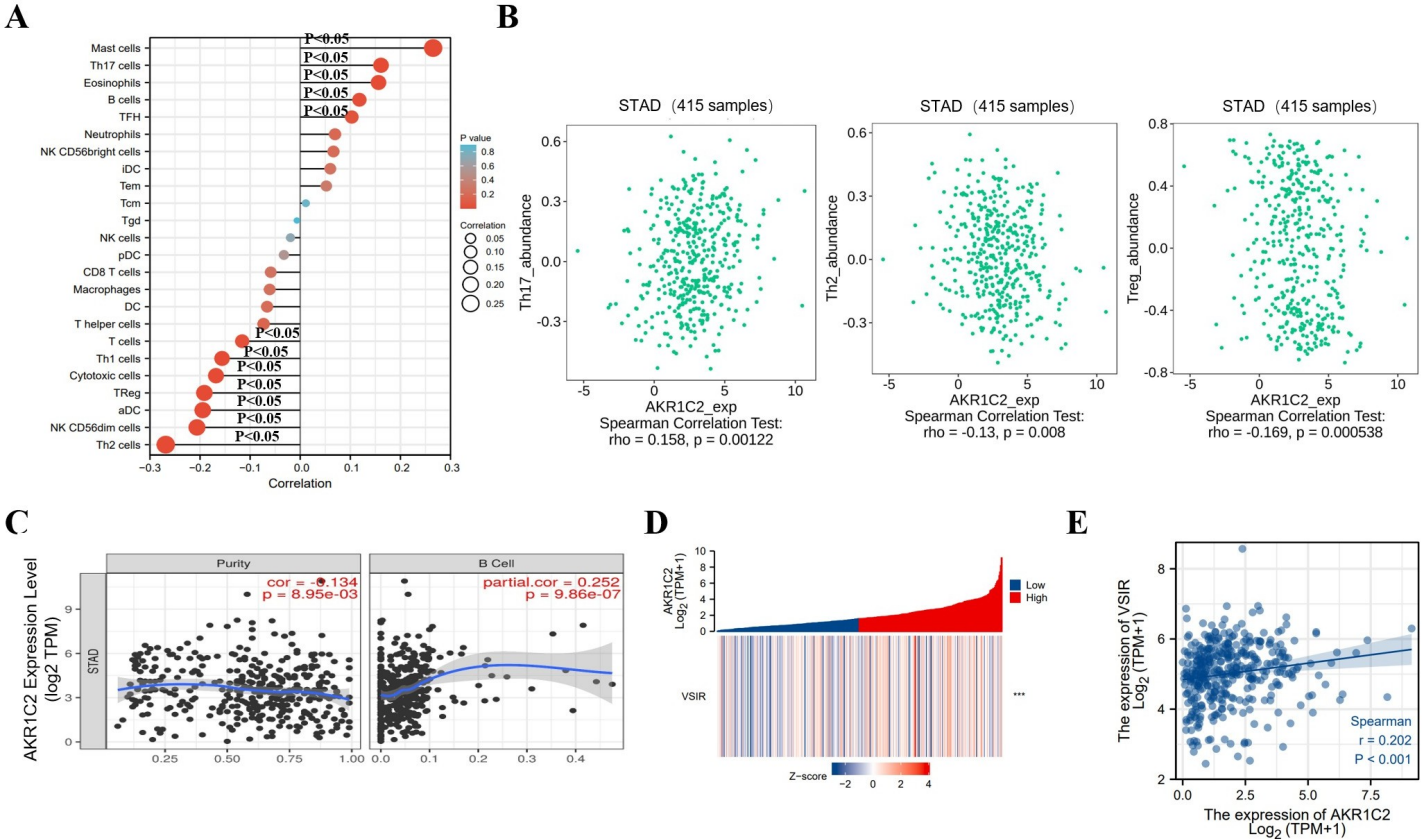
The link between AKR1C2 expression and immune infiltration of GC. **(A)** The correlation between AKR1C2 expression and 24 types of immune cells in TCGA database. **(B)** The scatterplot depicting the correlation between AKR1C2 and tumor-infiltrating lymphocytes (TILs). **(C)** The TIMER database showing the relationship between AKR1C2 and B cell. **(D-E)** The heatmap and scatterplot showing the correlation between the AKR1C2 and VSIR.

**Fig 6:**
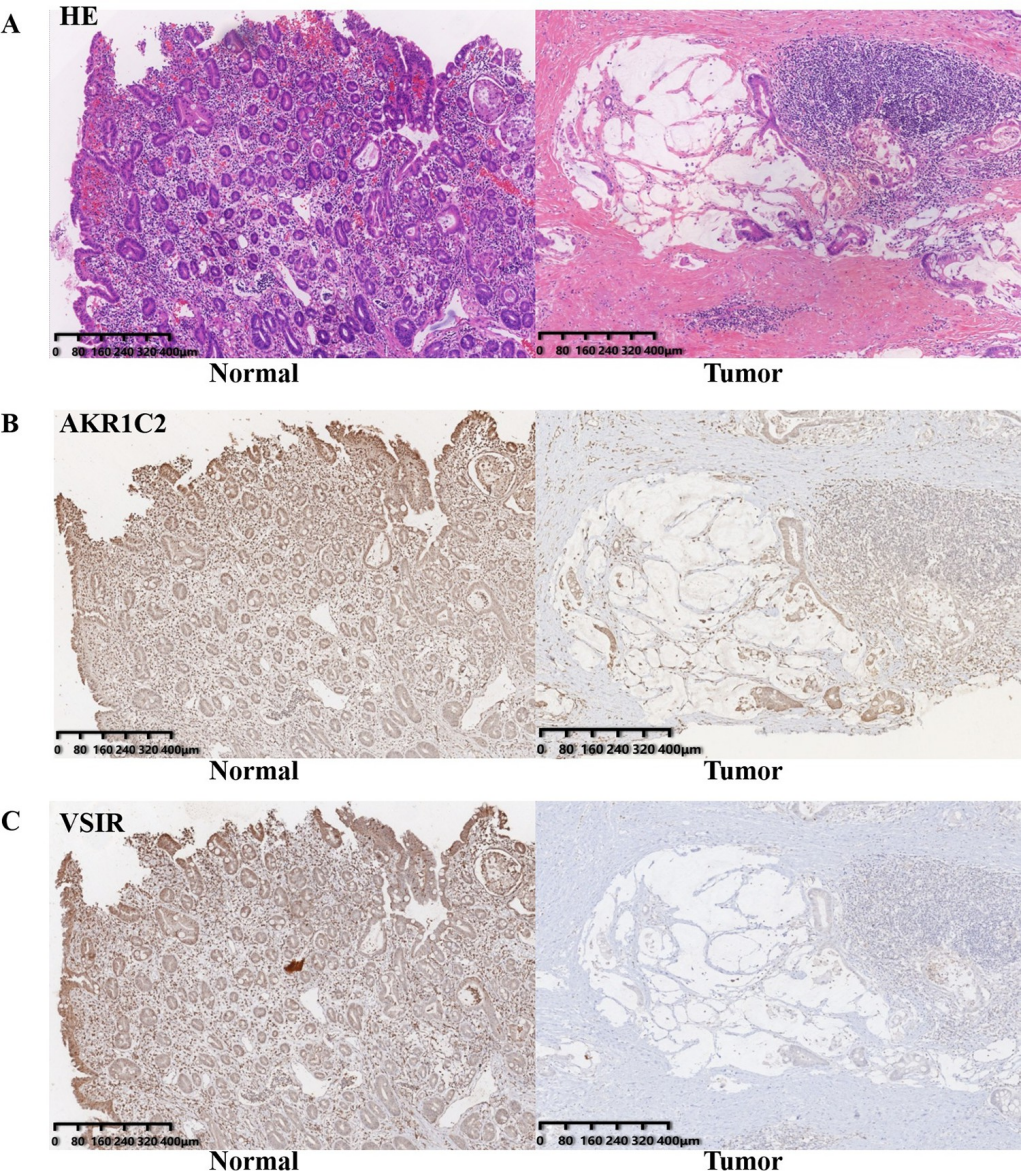
The expression levels of AKR1C2 and VSIR in the normal gastric tissues and GC tissues. **(A)** The picture showing the hematoxylin-eosin (HE) staining of the normal gastric tissues and GC tissues. **(B-C)** The immunohistochemical (IHC) analysis depicting upregulated AKR1C2 and VSIR in normal tissues.

In order to investigate the relationship between the expression level of AKR1C2 and immune infiltration, we applied the ssGSEA with Spearman correlation. As shown in **Fig 5A**, AKR1C2 expression was positively correlated with the infiltration of Mast cells, T helper type 17 (Th17) cells, Eosinophils, B cells and T follicular helper (Tfh) cells (p < 0.05). AKR1C2 expression was found to have negative correlation with infiltration of T helper type 2 (Th2) cells, NK CD56dim cells, activated DC (aDC), Treg, cytotoxic cells T helper type 1 (Th1) cells and T cells (p < 0.05). At the same time, by using TISIDB database, we further identified that AKR1C2 expression was positively correlated with Th17 cells infiltration and negatively correlated with infiltration of Th2 cells and Treg (**Fig 5B**). Also, the plot downloaded from TIMER database reflected that the expression level of AKR1C2 had positive correlation with B cells (**Fig 5C**). Moreover, through exploring the expression correlation between AKR1C2 and immune checkpoints, we found that AKR1C2 expression had positive correlation with VSIR (**Fig 5D and 5E**). In addition, IHC analysis was employed to explore the expression levels of AKR1C2 and VSIR in GC tissues and normal gastric tissues. **Fig 6A** portrayed the hematoxylin-eosin (HE) data of the normal and malignant gastric tissues. AKR1C2 and VSIR were both down-regulated in GC tissues (**Fig 6B and 6C**).

On the other hand, the correlation between AKR1C2 and immune regulation were further explored via some immune signatures of TISIDB database, like immunostimulators, immunoinhibitors, chemokines and receptors. The diagram indicated the correlation between AKR1C2 expression and immunostimulators of GC patients (**S2A Fig**). The top four positively correlated immunostimulators were HHLA2 (Spearman r = 0.296, p = 9.31e-10), C10orf54 (Spearman r = 0.229, p = 2.51e-06), RAET1E (Spearman r = 0.187, p = 0.000132) and TNFRSF13B (Spearman r = 0.119, p = 0.0157) (**S2B Fig**). The top four negatively AKR1C2-related immunoinhibitors included CTLA4 (Spearman r = −0.225, p = 1.6e-07), LAG3 (Spearman r = −0.223, p = 4.88e-06), CD274 (Spearman r = −0.217, p = 8.33e-06) and IDO1 (Spearman r = −0.203, p = 3.3e-05) (**S2C and S2D Fig**). In addition, **S3A Fig** demonstrated the correlation between AKR1C2 expression and chemokines. The top positively correlated molecules were CXCL14 (Spearman r = 0.302, p = 4.1e-10), CCL28 (Spearman r = 0.295, p = 1.01e-09), CXCL17 (Spearman r = 0.234, p = 1.52e-06) and CCL14 (Spearman r = 0.201, p = 3.96e-05) (**S3B Fig**). Furthermore, the correlation between AKR1C2 and receptors was exhibited in **S3C Fig**. Among all the related receptors, CCR8 (Spearman r = −0.212, p = 1.41e-05), CCR1 (Spearman r = −0.16, p = 0.00111), CCR5 (Spearman r = −0.153, p = 0.00177) and CXCR6 (Spearman r = −0.142, p = 0.00366) were receptors strongly negatively correlated with AKR1C2 (**S3D Fig**). Consequently, the results indicated that AKR1C2 was involved in the immune regulation of GC.

## Discussion

We investigated the new biomarkers involved in the process of ferroptosis in GC patients by using some bioinformatics tools. Through identifying the co-DEGs of ferroptosis-related gene dataset and three GEO datasets, we found two down-regulated genes AKR1C2 and MUC1 after analyzing the correlation between the two down-regulated genes and the prognosis of GC patients. The results revealed that the high expression levels of AKR1C2 were followed by a favorable prognosis in GC. We found that AKR1C2 was expressed higher in normal group than in GC group. Then, the plots showed that AKR1C2 participated in the progression of GC. Furthermore, this study paid more attention on whether AKR1C2 has the potential to be a predictive biomarker of GC progression. The LinkedOmics analysis demonstrated the co-expressed genes positively and negatively correlated with AKR1C2. Through ssGESA analysis, the figures exhibited that AKR1C2 participated in the biological functions of GC. Finally, the related immune analysis identified that AKR1C2 was involved in the immune regulation of GC patients.

Ferroptosis is a cell death form that is strongly correlated with lipid peroxidation and special modulators [26]. The cysteine metabolism and GPX4 inactivation have been proved to play important roles in ferroptosis [27]. Recent studies showed that it takes part in the process of some human diseases and cancers [28]. Certainly, ferroptosis is also correlated with GC progression. The clinical statistics illustrated that cancer-related fibroblasts (CAFs) induced the secretion of exosomal miR-522, thus inhibiting ferroptosis via ALOX15 and decreasing lipid-ROS in GC. The upregulation of fatty acid desaturase 1 (FADS1) and elongation of very long-chain fatty acid protein 5 (ELOVL5) in mesenchymal-type GC cells were found to cause ferroptosis sensitization. However, in intestinal-type GC cells, they may take part in the GC cells’ resistance to ferroptosis. SCD1 was identified to be able to induce cell proliferation and anti-ferroptosis of GC *in vivo* and *in vitro* [29]. Furthermore, ferroptosis may be initiated by MiR-375 through SLC7A11, which then causes the decrease of stemness of GC cells. Additionally, CDO1 is modulated by c-Myb, and the CDO1 silence can inhibit ferroptosis in GC cells. CDO1 suppression can prevent the generation of ROS and decrease malondialdehyde [30]. Moreover, perilipin2 facilitation plays a significant role in the cell growth and apoptosis of GC by modulating ferroptosis-related genes, such as acyl-coa synthetase long-chain family member 3, arachidonate 15-lipoxygenase, pr/set domain 11 and importin 7 [31]. These findings have proven that ferroptosis is vital in the progress of GC. However, the correlation of ferroptosis-related genes and the GC patients’ prognosis should be further explored and verified. This study investigated the significance of AKR1C2 in GC patients for the first time, and the results identified that high expression of this ferroptosis-related gene was correlated with a good prognosis.

A study indicated that AKR1C2 expression is correlated with fat distribution of human bodies [32], and AKR1C2 plays a vital role in the process of some cancers. A study also identified that AKR1C2 could be an oncogene in esophageal squamous cell carcinoma through mediating PI3K/AKT pathway [33]. The down-regulated expression level of AKR1C2 combined with upregulated expression of SRD5A1 and SRD5A3 may lead to the high levels of DHT either in primary or metastatic prostate cancer [34]. Meantime, AKR1C2 expression could be elevated by curcumin therapy to give play to the antitumor effects on prostate cancer [7]. These findings indicated that AKR1C2 expression was highly correlated with the cancer treatment. Similarly, we verified that the ferroptosis-related gene AKR1C2 was down-regulated in GC in this study.

Several statistics have identified that TIICs exert vital effects on the progression and clinical features of cancer [35]. In the treatment of patients with malignant tumors, immunotherapy has become a great strategy in the clinical employment. The delivery systems of immunotherapy should overcome barriers of minimal systemic toxicity [36]. Although the incidence rate of GC is decreasing these years, it still ranks third of all the cancer types. The prognosis of GC is poor for patients during late stages of cancer [37, 38]. Therefore, we should work together to investigate new therapies concerning GC patients. Immunotherapies of cancer include checkpoint inhibitors and chimeric cellular therapies [39]. A study reported that CXCL8, secreted by the macrophages, could trigger PD-L1^+^ macrophages and generate anticancer activity on GC cells [40]. CTLA4 is an immune-related receptor capable of suppressing T cell function in various types of cancers [41]. The mutations of CTLA4 are associated with lymphopenia and the reductions of tumor-infiltrating T cells, B cells, and natural killer (NK) cells [42]. This study evaluated the correlation between AKR1C2 and immune infiltration. The results revealed that AKR1C2 expression had positive correlation with Th17 cells and negative correlation with Th2 cells and Treg. Moreover, AKR1C2 was significantly associated with immunostimulators (HHLA2, C10orf54, RAET1E and TNFRSF13B), immunoinhibitors (CTLA4, LAG3, CD274 and IDO1), chemokines (CXCL14, CCL28, CXCL17 and CCL14) and chemokine receptors (CCR8, CCR1, CCR5 and CXCR6). Accordingly, several studies have also confirmed the important roles of aberrant AKR1C2 levels in the regulation of immune response in cancer patients. Lv et al. demonstrated that AKR1C2 could be served as an immune response-associated biomarker involving in the patients’ outcomes and immunotherapeutic effect [43]. To sum up, these results implied that AKR1C2 expression is significantly associated with immune response, and it has the promising potential to serve as an immunotherapeutic target for the treatment of GC.

Several studies reported that AKR1C2 has the prognostic values in some types of cancers. A recent study demonstrated that a signature consisting of five genes (AKR1C2, NCAN, AHCY, FBP2 and GALNT3) has potential prognostic values in neuroblastoma [44]. AKR1C2, as a risky gene, is correlated with the biochemical recurrence in prostate cancer patients after radical prostatectomy [43]. Moreover, down-regulated AKR1C2 was found to be correlated with the disease progression in patients with esophageal squamous cell carcinoma [45]. However, using Kaplan-Meier Plotter, we concluded that GC patients with down-regulated AKR1C2 favorable prognosis. These inconsistent results might be caused by the difference of genetic background or heterogeneity in patients with different tumors.

## Conclusion

In summary, this study illustrated that AKR1C2 was strongly correlated with prognosis of GC patients. Furthermore, the expression of AKR1C2 was correlated with immunostimulators, immunoinhibitors, chemokines and receptors. Therefore, the ferroptosis-related gene AKR1C2 has the potential to serve as a promising prognostic predictive biomarker for GC patients.

## Supporting information

S1 Fig Venn analysis depicted the upregulated co-DEGs between the ferroptosis related gene dataset and the three GEO datasets. There existed no up-regulated co-DEGs between the ferroptosis-related gene dataset and the three GEO datasets.
(TIF)

S2 Fig The relationship between AKR1C2 expression and immune signatures in GC. **(A)** The relationship between AKR1C2 expression and immunostimulators. **(B)** The top four immunostimulators that are positively associated with AKR1C2 expression. **(C)** The relationship between AKR1C2 expression and immunoinhibitors. **(D)** The top four immunoinhibitors that are negatively associated with AKR1C2 expression.
(TIF)

S3 Fig The relationship between AKR1C2 expression and chemokines or receptors in GC. **(A)** The relationship between AKR1C2 expression and chemokines. **(B)** The top four chemokines that are associated with AKR1C2 expression. **(C)** The relationship between AKR1C2 expression and receptors. **(D)** The top four receptors that are associated with AKR1C2 expression.
(TIF)

S1 Table The upregulated and downregulated genes of the three datasets obtained from GEO database. (XLS)

S2 Table The top 25 genes positively related with AKR1C2 in GC. (DOCX)

S3 Table The top 20 genes negatively related with AKR1C2 in GC. (DOC)

S4 Table Bioinformatic tools using to evaluate the significance of AKR1C2 in GC. (DOCX)
