# Supplementary figures and images for: Comprehensive analysis regarding the prognostic significance of downregulated ferroptosis-related gene AKR1C2 in gastric cancer and its underlying roles in immune response
Source: PLoS One. 2023 Jan 26;18(1):e0280989. doi: 10.1371/journal.pone.0280989 (PMC9879425; doi:10.1371/journal.pone.0280989)

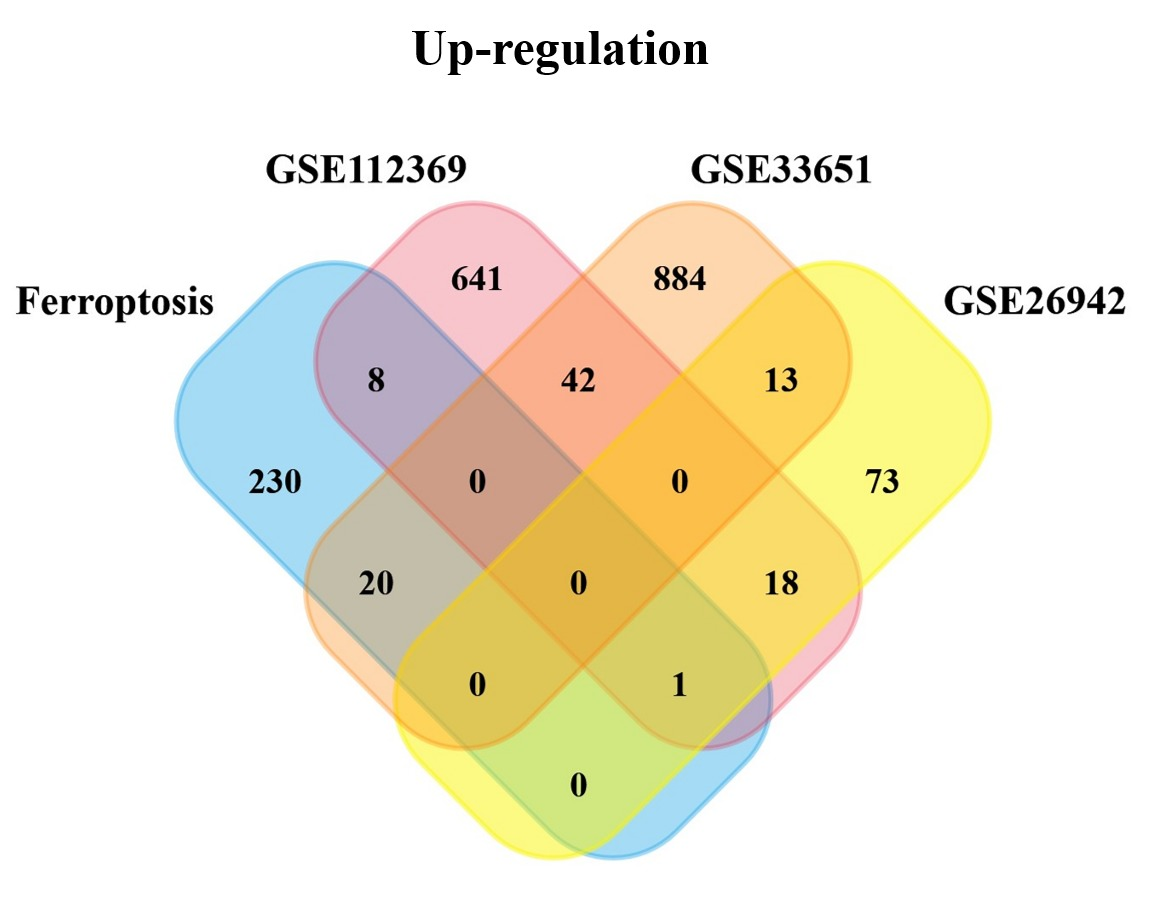

Supplement: S1 Fig — There existed no up-regulated co-DEGs between the ferroptosis-related gene dataset and the three GEO datasets. (TIF) [file pone.0280989.s001.tif]

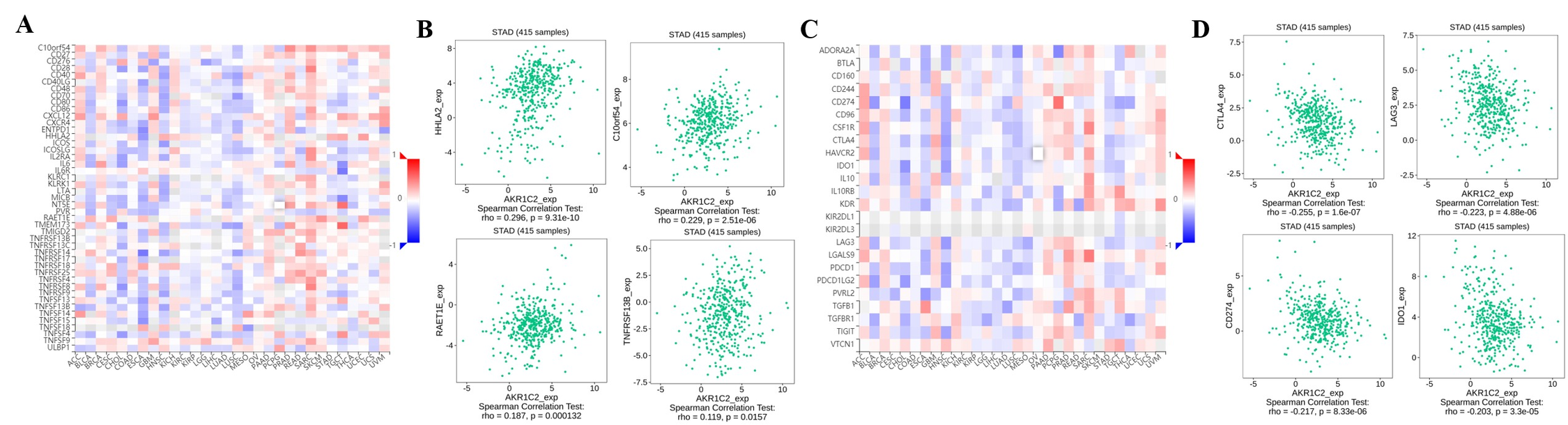

Supplement: S2 Fig — (A) The relationship between AKR1C2 expression and immunostimulators. (B) The top four immunostimulators that are positively associated with AKR1C2 expression. (C) The relationship between AKR1C2 expression and immunoinhibitors. (D) The top four immunoinhibitors that are negatively associated with AKR1C2 expression. (TIF) [file pone.0280989.s002.tif]

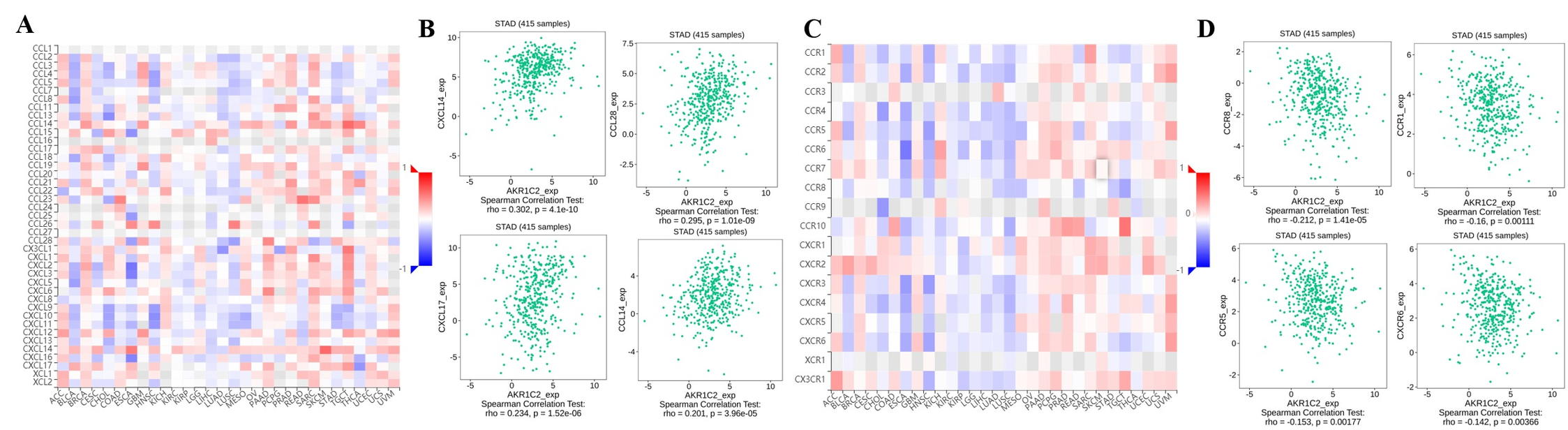

Supplement: S3 Fig — (A) The relationship between AKR1C2 expression and chemokines. (B) The top four chemokines that are associated with AKR1C2 expression. (C) The relationship between AKR1C2 expression and receptors. (D) The top four receptors that are associated with AKR1C2 expression. (TIF) [file pone.0280989.s003.tif]
