# Supplementary material for: Comprehensive analysis regarding the prognostic significance of downregulated ferroptosis-related gene AKR1C2 in gastric cancer and its underlying roles in immune response
Source: PLoS One. 2023 Jan 26;18(1):e0280989. doi: 10.1371/journal.pone.0280989 (PMC9879425; doi:10.1371/journal.pone.0280989)
Supplement: S2 Table — (DOCX) [file pone.0280989.s005.DOCX]

**Supplementary Table S2. The top 25 genes positively related with AKR1C2 in GC.**

| MYC | TTF1 | NDRG1 | STAT3 | ETS1 |
| --- | --- | --- | --- | --- |
| PKC | TP53 | GAPDH | ADAR | MYH9 |
| FOXM1 | MS4A1 | BCL2 | SERPINE1 | MYH11 |
| SCD1 | SFRS1 | IGFBP2 | MRE11A | ATM |
| CCNB1 | STAT5A | ARAF | FN1 | RBM15 |
