# Supplementary material for: Comprehensive analysis regarding the prognostic significance of downregulated ferroptosis-related gene AKR1C2 in gastric cancer and its underlying roles in immune response
Source: PLoS One. 2023 Jan 26;18(1):e0280989. doi: 10.1371/journal.pone.0280989 (PMC9879425; doi:10.1371/journal.pone.0280989)
Supplement: S3 Table — (DOC) [file pone.0280989.s006.DOC]

**Supplementary Table S3. The top 20 genes negatively related with AKR1C2 in GC.**

| ITGA2 | EIF4E | CTNNB1 | FASN |
| --- | --- | --- | --- |
| COL6A1 | BID | ERCC1 | NFKB1 |
| SRC | PXN | MAPK8 | BAP1 |
| MAPK14 | NOTCH1 | STMN1 | ACVRL1 |
| EIF4EBP1 | CDH1 | EEF2 | IRS1 |
