# Supplementary material for: Comprehensive analysis regarding the prognostic significance of downregulated ferroptosis-related gene AKR1C2 in gastric cancer and its underlying roles in immune response
Source: PLoS One. 2023 Jan 26;18(1):e0280989. doi: 10.1371/journal.pone.0280989 (PMC9879425; doi:10.1371/journal.pone.0280989)
Supplement: S4 Table — (DOCX) [file pone.0280989.s007.docx]

**Supplementary Table S4. Bioinformatic tools using to evaluate the significance of AKR1C2 in GC.**

| Database | URL | Refs |
| --- | --- | --- |
| GEO | https://www.ncbi.nlm.nih.gov/gds/?term= | [11] |
| TCGA | https://portal. gdc.cancer.gov/ | [15] |
| Kaplan-Meier Plotter | http://kmplot.com/analysis/ | [16] |
| TNMplot | [http://www.tnmplot.com](http://www.tnmplot.com/) | [17] |
| LinkedOmics | http://www.linkedomics.org/admin.php | [19] |
| TISIDB | http://cis.hku.hk/TISIDB/ | [23] |
| TIMER | https://cistrome.shinyapps.io/timer/ | [24] |
| GEPIA2.0 | http://gepia.cancer-pku.cn/ | [18] |
